# Interventional Vitamin Mix Glaucoma Study (IVMGS): study protocol for a prospective, randomized, two-arm, single-center trial in existing glaucoma patients

**DOI:** 10.1186/s13063-025-09168-z

**Published:** 2025-10-13

**Authors:** Navid Golpour, Flora Hui, Maria Nilsson, Jonas Svensson, Rune L. Brautaset, James R. Tribble, Pete A. Williams

**Affiliations:** 1 Department of Clinical Neuroscience, Division of Eye and Vision, St. Erik Eye Hospital, Karolinska Institutet, Stockholm, Sweden; 2 Centre for Eye Research Australia, Royal Victorian Eye and Ear Hospital, Melbourne, Australia; 3 Department of Clinical Neuroscience, Karolinska Institutet and Stockholm Health Care Services, Region Stockholm, Stockholm, Sweden

**Keywords:** Glaucoma, Neuroprotection, One-carbon metabolism, Vitamins B6, B9, B12, Coline, Electroretinography, Photopic negative response, Randomized controlled trial, Protocol

## Abstract

**Background:**

Glaucoma is a leading cause of irreversible blindness, characterized by progressive degeneration of retinal ganglion cells. Current treatments primarily lower intraocular pressure but do not directly provide neuroprotection. Preclinical studies from our group have identified dysfunction in one-carbon metabolism as a contributor to glaucomatous neurodegeneration in rodent models. The Interventional Vitamin Mix Glaucoma Study will evaluate whether 12 months of supplementation with key one-carbon metabolism cofactors and precursors (vitamins B_6_, B_9_, B_12_, and choline) can improve inner retinal function and provide neuroprotection compared with standard care alone.

**Methods:**

The Interventional Vitamin Mix Glaucoma Study is a Phase 2a, open-label, randomized, two-arm clinical trial. Participants will be assigned in a one-to-one ratio to receive either daily one-carbon metabolism supplementation plus standard care or standard care alone. The study will enroll 80 patients with primary open-angle glaucoma, normal tension glaucoma, or pseudoexfoliation glaucoma, each with mild-to-moderate visual field loss in at least one eye. Recruitment will take place from March 2025 to March 2026 at St. Erik Eye Hospital, Karolinska Institutet, Stockholm, Sweden. The primary outcome is change in the photopic negative response measured by full-field electroretinography. Secondary outcomes include changes in visual field parameters, retinal nerve fiber layer thickness, and ganglion cell–inner plexiform layer thickness. Exploratory outcomes will include changes in blood-based metabolomic and DNA methylation profiles.

**Discussion:**

One-carbon metabolism–based supplementation has been shown to reduce retinal ganglion cell loss in rodent models of glaucoma. This trial will investigate whether such supplementation improves retinal function and confers neuroprotection in patients with glaucoma. The use of full-field electroretinography as the primary outcome aims to detect early functional improvements in the retina. If successful, this approach could support a low-cost, widely available neuroprotective strategy to be used alongside existing intraocular pressure–lowering therapies.

**Trial registration:**

This trial is registered at ClinicalTrials.gov (NCT06885827), first posted on 20 March 2025.

**Supplementary Information:**

The online version contains supplementary material available at 10.1186/s13063-025-09168-z.

## Introduction

### Background and rationale {6a}

Glaucoma is a neurodegenerative disease and the leading cause of irreversible blindness worldwide [1]. It is characterized by progressive dysfunction and loss of retinal ganglion cells (RGCs) and their axons in the optic nerve. Age, genetics, and high intraocular pressure (IOP) are the main risk factors for glaucoma. Current treatments focus on lowering IOP, which is effective in approximately 60% of patients. However, it does not directly target neuroprotection of RGCs, and up to 42% of treated patients are expected to lose vision in at least one eye [2]. This suggests that the degeneration of RGCs is multifactorial, with the need for treatments targeting additional mechanisms of disease progression.

Metabolic dysfunction, particularly mitochondrial abnormalities, plays a crucial role in glaucoma pathophysiology. The unmyelinated axons of retinal ganglion cells (RGCs) at the lamina cribrosa in the optic nerve head (ONH) have the highest mitochondrial density, making them highly susceptible to metabolic stress [3]. Metabolic dysfunction has emerged as a critical insult in glaucoma pathophysiology, and as such, targeting metabolism may provide an attractive therapeutic option [4]. A promising example of this approach is nicotinamide treatment. High-dose supplementation of nicotinamide has been successfully translated to two positive Phase II clinical trials following the identification and development of this treatment in rodent models [5–9]. A significantly improved retinal function, as measured by full-field electroretinogram (ffERG), was demonstrated in nicotinamide-treated participants, relative to untreated, in as little as 12 weeks [8]. Exploring further metabolic dysfunctions in glaucoma, we recently identified elevated homocysteine and early and sustained dysregulation of genes consistent with one-carbon metabolism (1CM) dysfunction in glaucoma animal models and human iPSC RGCs [10]. This dysregulation extends to the transport and utilization of essential 1CM cofactors and precursors (vitamins B_6,_ B_9_, B_12_, and choline) in the retina and optic nerve head. Rodents induced with experimental glaucoma were supplemented with B_6_, B_9_, B_12_, and choline in both an acute and chronic model of glaucoma. Results demonstrated a significant reduction in RGC loss in an IOP-independent manner in both acute and chronic models. Markers of optic nerve health and RGC specific components of the ERG were significantly protected in 1CM vitamin-treated animals. The results suggest that these 1CM-based treatments may offer neuroprotection in glaucoma. Given the availability and safety profile of these supplements, this IVMGS trial aims to investigate the effectiveness of B_6_, B_9_, B_12_, and choline supplementation on retinal function in existing glaucoma patients, primarily evaluated by ffERG, over a 12-month period. The hypothesis is that the 1CM-based supplementation can improve retinal function and slow disease progression.

### Objectives {7}

#### Primary objective

To determine whether 12 months of oral one-carbon metabolism (1CM) supplementation, comprising vitamins B6, B9, B12, and choline improves inner retinal function, measured by photopic negative response (PhNR) amplitude (an RGC specific component) on full-field electroretinography (ffERG), in patients with glaucoma compared with standard care alone.

#### Secondary objectives

To assess whether 1CM supplementation affects visual field parameters and retinal nerve fiber layer (RNFL) and ganglion cell–inner plexiform layer (GCL–IPL) thickness measured by optical coherence tomography (OCT).

## Hypothesis

One-carbon metabolism cofactor and precursor (vitamins B_6_, B_9_, B_12_, and choline) supplementation will significantly improve inner retinal function, as evidenced by increased PhNR amplitude, compared with standard care alone in patients with glaucoma.

### Trial design {8}

The Interventional Vitamin Mix Glaucoma Study is a 12-month, investigator-initiated, Phase 2a, open-label, randomized controlled trial with two parallel groups in a one-to-one allocation ratio. The trial has a superiority framework, comparing standard care alone with standard care plus daily supplementation of vitamin B_6,_ B_9_, B_12_, and choline. The unit of randomization is the individual participant, who will be randomly assigned to either group. Follow-up visits will take place at baseline, 3, 6, 9, and 12 months, as detailed in Table 1.

**Table 1:** Baseline visit, clinical follow-ups, and their corresponding examinations

**Procedure**	**Pre-screening and inclusion**	**Baseline visit (0)**	**Visit 1,** **3 months** **(± 14 days)**	**Visit 2,** **6 months** **(± 14 days)**	**Visit 3,** **9 months** **(± 14 days)**	**Visit 4,** **12 months** **(± 14 days)**
Check inclusion/exclusion criteria	√					
Written consent	√					
Randomization	√					
Health declaration	√					
AR		√				
VA		√	√	√	√	√
IOP		√	√	√	√	√
OCT		√	√			√
VF		√	√			√
ffERG		√	√			√
Blood collection		√	√			√
Follow-up questionnaire			√	√	√	√
Study end						√

*AR *auto refraction, *VA* visual acuity, *IOP* intraocular pressure, *OCT* optical coherence tomography, *VF* visual field, *ffERG* full-field electroretinogram

## Methods: participants, interventions, and outcomes

### Study setting {9}

This single-center trial will be conducted at St. Erik Eye Hospital, an academic hospital in Stockholm, Sweden, in collaboration with the Department of Clinical Neuroscience (Division of Eye and Vision) at Karolinska Institutet. All participant recruitment, study visits, and data collection will be performed at St. Erik Eye Hospital.

### Eligibility criteria {10}

#### Inclusion criteria

- Age ≥ 18 years
- Primary open-angle glaucoma, normal tension glaucoma, or pseudoexfoliation glaucoma in one or both eyes diagnosed by an ophthalmologist
- Best-corrected Snellen visual acuity of 0.3 or better in the study eye(s)
- Two or more reliable visual field tests with fewer than 15% false positives

#### Exclusion criteria

- Visual field damage worse than − 16 dB in the study eye(s)
- Intraocular pressure greater than 35 mmHg in the study eye(s) on either of two measurement occasions, or a mean pressure of 30 mmHg over two occasions
- Any disease affecting retinal function
- Neurological or other non-glaucomatous conditions that may affect the visual field
- Inability to perform visual field examination
- Unwillingness to stop intake of multivitamins or B vitamin substances
- Known allergy or intolerance to B vitamins
- Previous eye surgery, except for uncomplicated cataract surgery
- Pregnant or breastfeeding women
- Women of childbearing potential who do not use reliable contraception
- Any disease or condition likely to prevent long-term follow-up
- Cancer diagnosis within the last 5 years (except treated squamous cell carcinoma)
- History of liver disease or stomach ulcers
- Inability to understand and speak Swedish or English

### Who will take informed consent? {26a}

Licensed medical doctors at St. Erik Eye Hospital will identify eligible participants, provide written study information, and allow at least 24 h for consideration. At the baseline visit, before any study-specific procedures, a recruiting physician will discuss the trial, answer questions, and obtain written informed consent from the participant, with both parties signing the form and completing a health declaration on site.

### Additional consent provisions for collection and use of participant data and biological specimens {26b}

Consent for collection of biological specimens is included in the main trial informed consent. Participants agree to provide blood samples at baseline, 3 months, and 12 months for analyses related to this trial only. Samples will be stored in the Stockholm Medical Biobank (registration number 914) in accordance with the Swedish Biobank Act (2023:38) and destroyed after study completion. No use for ancillary or future studies is planned without obtaining additional ethical consent.

## Interventions

### Explanation for the choice of comparators {6b}

Standard care, as prescribed by the treating ophthalmologist (primarily intraocular pressure–lowering eye drops), was chosen because it reflects current best practice for glaucoma management in Sweden. With no approved neuroprotective treatments available, standard care without vitamin supplementation provides an appropriate control.

### Intervention description {11a}

#### Control arm

Participants will receive standard care only (for 12 months), consisting of glaucoma management as determined by the treating ophthalmologist, typically intraocular pressure–lowering eye drops. No one-carbon metabolism supplementation will be provided.

#### Intervention arm

Participants will receive standard care plus daily oral one-carbon metabolism supplementation for 12 months, starting at the baseline visit after randomization. The regimen comprises:

- Vitamin B_6_ (pyridoxine): 25 mg, 1 capsule daily
- Vitamin B_9_ (folic acid): 400 μg, 2 capsules daily (800 μg total), which also contain Vitamin B_12_
- Vitamin B_12_ (cyanocobalamin): 1 mg, included in Vitamin  B_9_ capsules (2 mg total)
- Choline (choline bitartrate): 500 mg, 2 capsules daily (1000 mg total)

All capsules will be taken orally once daily in the morning with a full glass of water and food. The intervention is self-administered at home. No tailoring of dose or schedule is planned.

#### Dosing rationale

The regimen was based on a preclinical glaucoma model in which the tested cofactors and precursors reduced retinal ganglion cell loss [10]. Rodent doses were converted to human-equivalent doses using allometric scaling and adjusted to remain below adult tolerable upper intake levels (Table 2).

**Table 2:** HED, safety data, and drug formulation for 1CM-cofactors and precursors

Compound	Rodent dose	Equivalent human dose (HED)	For 70 kg human	Tolerable upper intake level (UL)	Formulation
B_6_ (as pyridoxine)	4.5 mg/kg	0.72 mg/kg	50.4 mg/day	100 mg/day	1 × 25 mg capsules
B_9_ (as folic acid)	1.5 mg/kg	0.25 mg/kg	16.8 mg/day	1 mg/day	2 × 0.4 mg capsule
B_12_ (as cyanocobalamin)	20 μ/kg	3.2 μ/kg	0.224 mg/day	No limit orally due to limit of bioavailability	2 mg included in folic acid capsule
Choline (as choline bitartrate)	750 mg/kg	80 mg/kg	5.6 g/day	3.5 g/day	2 × 500 mg capsules

### Criteria for discontinuing or modifying allocated interventions {11b}

Supplementation may be discontinued if participants experience intolerable adverse events, request to withdraw, or show significant disease progression despite standard care. Discontinuation will also occur if safety endpoints are met, defined as deterioration of ≥ 3 points at the same location relative to baseline on Glaucoma Progression Analysis (GPA) software (probability of change < 0.05) on three consecutive visits, and/or intraocular pressure > 30 mmHg on two consecutive visits.

### Strategies to improve adherence to interventions {11c}

Adherence will be assessed at each follow-up visit by capsule counts and participant logs. Compliance will be calculated as the proportion of doses taken, with ≥ 80% considered acceptable. Participants with lower compliance will remain in the trial for intention-to-treat analysis but will be excluded from the per-protocol analysis.

### Relevant concomitant care permitted or prohibited during the trial {11d}

Participants may continue any glaucoma treatment prescribed by their ophthalmologist. Initiation of new vitamin B_6_, B_9_, B_12_, or choline supplements, including multivitamins containing these or other B vitamins, is prohibited during the study period. Other concomitant treatments unrelated to the study intervention are permitted.

### Provisions for post-trial care {30}

No specific post-trial care is planned beyond standard glaucoma management by the treating ophthalmologist. The vitamins used in the study are available over the counter in Sweden. Participants will not be advised to start the treatment based on the trial outcome but those who wish to continue (or start) supplementation can be advised on the regimen applied in the trial. They will also be informed that long-term safety and efficacy in relation to glaucoma have not been established. Participants are covered by the Swedish national patient injury insurance (Patientskadelagen) for any harm resulting from trial participation.

### Outcomes {12}

#### Primary outcome

The primary outcome is inner retinal function, measured by full-field electroretinography (ffERG) photopic negative response (PhNR) using PhNR Vmax and the PhNR/b-wave ratio. The main analysis will assess change from baseline to 12 months, with 3-month values summarized descriptively and included in longitudinal models. Results will be reported as group mean change with 95% confidence intervals. Assessments occur at baseline, 3 months, and 12 months. PhNR is a marker of RGC function and can detect early neurofunctional changes in glaucoma.

#### Secondary outcomes

1. Visual field sensitivity will be measured using Humphrey 24–2 SITA FAST, reported as mean deviation (MD), pattern standard deviation (PSD), and visual field index (VFI). The main analysis will assess change from baseline to 3 and 12 months, with results presented as group mean change with 95% confidence intervals. Visual field outcomes reflect functional progression in glaucoma.
2. Retinal structure will be evaluated with spectral-domain OCT (SD-OCT), measuring average and sectoral retinal nerve fiber layer (RNFL) thickness and ganglion cell–inner plexiform layer (GCL–IPL) thickness. Changes from baseline to 3 and 12 months will be reported as group mean change with 95% confidence intervals. OCT outcomes provide structural correlates of retinal ganglion cell loss.
3. Intraocular pressure (IOP) will be measured with the iCare IC100 tonometer, and visual acuity (VA) will be assessed using ETDRS charts and reported in logMAR units. The main analysis will assess change from baseline to 12 months, with interim values summarized at each visit. Results will be presented as group mean change with 95% confidence intervals. Assessments will occur at baseline, 3, 6, 9, and 12 months. IOP reflects disease control, and VA reflects overall visual function.

#### Exploratory outcomes

Blood metabolomics will be analyzed by high-resolution mass spectrometry (HRMS), and global DNA methylation will be assessed in peripheral blood mononuclear cells (PBMC) using LINE-1 5-mC/5-hmC analysis. The main analysis will compare profiles between groups at 12 months and, where applicable, assess change from baseline. Data will be summarized using multivariate and group-level analyses with 95% confidence intervals where suitable. Assessments will be performed at baseline, 3 months, and 12 months. These measures will explore systemic signatures of one-carbon metabolism.

#### Safety outcomes

Adverse events (AEs) and serious adverse events (SAEs) related to supplementation or study procedures will be recorded at each visit from baseline to 12 months and reported as the number of events and the proportion of participants affected, categorized by severity and relatedness.

### Participant timeline {13}

Eligible participants will be enrolled after screening and informed consent, with baseline assessments performed prior to randomization. Interventions and follow-up assessments will occur at baseline, 3, 6, 9, and 12 months. No run-in or washout period is planned. The schedule of enrolment, interventions, and assessments is shown in Table 1.

### Sample size {14}

Based on previous ffERG PhNR data following IOP lowering or nicotinamide treatment [8, 11], the sample size was calculated for a two-arm parallel design with *α* = 0.05, 80% power, two-sided hypothesis, and effect size (Cohen’s *d*) of 0.577. This requires 48 eyes per arm (96 total). Allowing for 20% drop-out increases the requirement to 120 eyes. With 70% of participants expected to have bilateral glaucoma (average 1.7 eyes/participant) and assuming an intra-class correlation of 0.20, the design effect is 1.14, giving a final requirement of approximately 137 eyes, corresponding to 82 participants (41 per arm).

### Recruitment {15}

Potential participants will be recruited via referrals from ophthalmologists at St. Erik Eye Hospital and private eye clinics. Recruitment is planned to start in March 2025 and continue until the target sample size has been reached (randomized), anticipated by March 2026. Each participant will be followed for 12 months from baseline to complete all primary and secondary outcome assessments.

## Assignment of interventions: allocation

### Sequence generation {16a}

The random allocation sequence will be generated by an independent researcher not involved in participant recruitment, assessment, or intervention delivery. A computer-generated random number sequence will be created in REDCap using variable block sizes of 2–4. No stratification factors will be used. The block sizes and full sequence file will be stored in a secure location, inaccessible to all other study personnel.

### Concealment mechanism {16b}

Central allocation concealment will be maintained via the REDCap randomization module. Study coordinators will access the randomization function only after confirming eligibility and obtaining informed consent. REDCap will release the group assignment at the point of randomization, ensuring that the next allocation in the sequence cannot be known in advance by anyone involved in enrolment.

### Implementation {16c}

The independent researcher will generate and upload the allocation sequence to REDCap. Study coordinators will enroll participants and initiate randomization within REDCap, which will assign participants according to the concealed sequence. The allocation list will not be available to investigators, study coordinators, or outcome assessors at any stage.

## Assignment of interventions: blinding

### Who will be blinded {17a}

Outcome assessors and biostatisticans will be blinded to group allocation. Blinding will be maintained by coding participant identifiers and removing allocation information from datasets provided for analysis.

### Procedure for unblinding if needed {17b}

Unblinding will be permitted only if knowledge of allocation is essential for the clinical management of a serious adverse event. The principal investigator will authorize unblinding, which will be performed by an independent researcher not involved in outcome assessment or analysis. The reason, date, and personnel involved will be recorded.

## Data collection and management

### Plans for assessment and collection of outcomes {18a}

Baseline data will include demographics, medical and ocular history, and baseline values for all outcome measures. Follow-up assessments will be performed at 3, 6, 9, and 12 months according to the trial schedule. Standardized protocols and calibrated equipment will be used for ETDRS visual acuity, Humphrey 24–2 visual field testing, SD-OCT imaging, and ffERG with PhNR analysis. Duplicate measurements will be taken for IOP, visual fields, and OCT to promote data quality.

At each study visit, all assessments will be performed bilaterally, even if only one eye meets the inclusion criteria. Visual field testing and tonometry will be conducted on undilated pupils, whereas ffERG and OCT will be performed after pharmacological pupil dilation with 0.5% tropicamide. A detailed schedule of assessments is provided in Table 1.

#### Assessment procedures

##### Refraction

Two measurements will be obtained using the auto refractometer Tonoref III (NIDEK, Gamagori, Aichi, Japan) to determine the average sphere, cylinder, and axis corrections.

##### Visual acuity

Measured with an Early Treatment Diabetic Retinopathy Study (ETDRS) chart at 4 m, with correction based on autorefraction readings. Threshold measurements will be used to calculate logMAR scores.

##### Tonometry

IOP will be measured before dilation using the iCare IC100 rebound tonometer. Six readings will be averaged automatically by the device. Two consecutive averaged measurements will be taken with the participant seated in primary gaze. A fluctuation of ± 2 mmHg is acceptable—otherwise, additional measurements will be taken.

##### Visual field testing

Automated perimetry will be performed using the Humphrey Field Analyzer 3 (Carl Zeiss Meditec, Dublin, CA, USA) and the 24–2 SITA FAST program to obtain Visual Field Index (VFI), mean deviation (MD), and pattern standard deviation (PSD). Each eye will be tested twice. Reliable tests require < 15% false positives.

##### Optical coherence tomography

Spectral-domain OCT will be conducted using the Cirrus OCT 5000 (Carl Zeiss Meditec). Peripapillary retinal nerve fiber layer (RNFL) thickness will be measured with the Optic Disc Cube 200 × 200 protocol, and ganglion cell layer plus inner plexiform layer (GCL + IPL) thickness with the Macular Cube 512 × 128 protocol. Both protocols will be performed twice per eye. Only high-quality images (signal strength > 6) will be used. Average and sectoral RNFL and GCL + IPL thicknesses will be recorded in micrometers.

##### Electroretinogram

Full-field photopic electroretinograms with photopic negative response (PhNR) will be recorded using the E3 Console with ColorBurst™ handheld ganzfeld stimulator and Espion V6 system (Diagnosys LLC, Lowell, MA). Subjects will be light-adapted in room light for at least 10 min and then adapted to the blue background of the stimulator for 1–2 min before each eye is tested. DTL-Plus electrodes will be used with a forehead gold-cup ground. Red flashes (0.07–12.56 cd·s/m^2^: 32 steps, 16 per eye) will be delivered on a blue background at 2 Hz. Signals will be sampled at 2000 Hz with a recording window of 20 ms pre- and 150 ms post-stimulus. At least 24 artifact-free sweeps will be averaged per step. a-wave, b-wave, and PhNR amplitudes and implicit times will be analyzed, and intensity–response functions will be modeled with a saturating hyperbolic function to determine Vmax.

##### Blood sampling

At the first, second, and final visits, two 10-ml venous blood samples (one EDTA tube, one serum tube) will be collected after a ≥ 3-h fast. Samples will be centrifuged after 30 min: plasma, buffy coat, erythrocytes, and serum will be aliquoted into 1-ml cryovials and stored at − 80 °C at St. Erik Eye Hospital before biobanking. Metabolomics will be analyzed using high-resolution mass spectrometry (HRMS). Global DNA methylation will be assessed from peripheral blood mononuclear cells (PBMCs) using commercial genomic DNA isolation kits, without recording identifiable genetic data. DNA methylation (5-methylcytosine, 5-mC) in LINE-1 repeats and hydroxymethylation (5-hydroxymethylcytosine, 5-hmC) will be quantified using commercial laboratory kits.

### Plans to promote participant retention and complete follow-up {18b}

All participants will be reminded of upcoming visits by sms and email. Outcome data will be collected for all participants who discontinue or deviate from the intervention protocol, including primary and secondary outcomes at all subsequent scheduled visits where possible.

### Data management {19}

Data will be entered into REDCap with built-in range checks and audit trails. All data will be de-identified, coded, and stored securely with access restricted to authorized study personnel. OCT and ERG data will be stored on secure physical media in a locked fireproof cabinet. The code linking personal identifiers to study IDs will be kept separately at the study site. Data from patients who leave the trial early will be stored as for other participants.

### Confidentiality {27}

Personal information will be collected through electronic medical records and case report forms. All data will be de-identified at collection, with a codebook linking personal identification numbers to trial IDs stored separately at the study site. Access to identifiable data will be restricted to authorized study personnel. OCT and ERG imaging data will be stored on secure media in a fireproof locker at Karolinska Institutet. Data will be retained securely before, during, and after the trial in accordance with Swedish data protection regulations.

### Plans for collection, laboratory evaluation and storage of biological specimens for genetic or molecular analysis in this trial/future use {33}

Venous blood will be collected at baseline, 3 months, and 12 months. Samples will be processed into plasma, buffy coat, erythrocytes, and serum, aliquoted, and stored at − 80 °C in the St. Erik Eye Hospital before storage in the Stockholm Medical Biobank. Metabolomics will be performed using high-resolution mass spectrometry, and global DNA methylation will be assessed from PBMCs using commercial kits without recording identifiable genetic data. No genetic sequencing will be performed. Samples will be stored for future use in ancillary studies approved by relevant ethics committees.

## Statistical methods

### Statistical methods for primary and secondary outcomes {20a}

The primary and secondary outcome measures will be compared between the treatment and control groups using mixed-effects linear regression models, with eye nested within participant to account for correlation between eyes. Analyses will be conducted under the intention-to-treat principle, with a per-protocol analysis including only participants achieving ≥ 80% adherence and no major protocol deviations. Statistical significance will be set at *α* = 0.05. Analyses will be performed in R (version 4.2.0 or later) or SPSS (version 28.0 or later). No adjustments for multiplicity are planned for secondary outcomes.

### Interim analyses {21b}

No interim analyses are planned. The trial will not have stopping guidelines for efficacy or futility. Only safety data will be reviewed periodically by the principal investigator and the study team to monitor for unexpected adverse events.

### Methods for additional analyses (e.g., subgroup analyses) {20b}

Exploratory analyses will include metabolomic and DNA methylation data, analyzed using principal component analysis (PCA) and partial least squares discriminant analysis (PLS-DA). Additional subgroup analyses may be conducted by glaucoma subtype (POAG, NTG, PEX) and baseline disease severity (mild vs. moderate) using similar mixed-effects regression models.

### Methods in analysis to handle protocol non-adherence and any statistical methods to handle missing data {20c}

The intention-to-treat (ITT) population will include all randomized participants, regardless of protocol adherence or study completion. The per-protocol (PP) population will include participants who complete the study without major protocol deviations and with ≥ 80% adherence to the intervention regimen. Missing outcome data will be handled using multiple imputation under the missing-at-random assumption, and sensitivity analyses will compare results with complete-case analyses.

### Plans to give access to the full protocol, participant-level data, and statistical code {31c}

The full protocol is publicly accessible via the ClinicalTrials.gov registry (NCT06885827). Participant-level data will not be made publicly available due to privacy and regulatory considerations in accordance with the EU General Data Protection Regulation (GDPR) and the Swedish Data Protection Act. Requests for access to the data can be directed to the Research Data Office (rdo@ki.se) at Karolinska Institutet. Statistical analysis code used for the trial’s primary and secondary analyses can be requested directly from the corresponding authors.

## Oversight and monitoring

### Composition of the coordinating center and trial steering committee {5d}

The trial is coordinated by the Department of Clinical Neuroscience, Division of Eye and Vision, St. Erik Eye Hospital, Karolinska Institutet. The Principal Investigator, Rune Brautaset, has overall oversight, while the clinical lead, Navid Golpour, is responsible for participant recruitment and operational management of the trial. Co-investigators contribute to trial design, conduct, and data analysis. The steering committee (Navid Golpour, Flora Hui, Maria Nilsson, James Tribble, Pete Williams, Rune Brautaset) meets quarterly to review safety data and overall study conduct.

### Composition of the data monitoring committee, its role and reporting structure {21a}

No independent data monitoring committee will be established because the intervention is considered low risk and the trial duration is limited. Safety monitoring will be performed by the principal investigator and study team, who will review adverse events on an ongoing basis.

### Adverse event reporting and harms {22}

At 3-, 6-, 9-, and 12-month visits, participants will be asked about any adverse events (AEs) and are encouraged to report such events directly to the trial coordinators at any time. All events are documented in the case report form and assessed for causality, intensity, and seriousness in accordance with ICH-GCP (E6 R2) definitions. Serious adverse events (SAEs) are defined as any untoward medical occurrence that results in death, is life-threathening, requires hospitalization, results in persistent disability, or is otherwise medically significant, and will be reported to the Principal Investigator within 24 h. All harms will be summarized and reported in the trial publication without restriction, in accordance with CONSORT guidelines.

### Frequency and plans for auditing trial conduct {23}

No formal independent audit of trial conduct is planned, given the single-center, low-risk nature of the study. The trial may be monitored or inspected at any time by Karolinska Institutet.

### Plans for communicating important protocol amendments to relevant parties (e.g., trial participants, ethical committees) {25}

Any important protocol modifications, including changes to eligibility criteria, outcomes, or analyses, will be submitted for approval to the Swedish Ethical Review Authority before implementation, updated on ClinicalTrials.gov, and communicated to all investigators and relevant trial personnel. If applicable, participants will be informed directly.

### Dissemination plans {31a}

Trial results will be communicated through publication in peer-reviewed scientific journals and presentation at scientific meetings. A lay summary will be provided to participants upon request after publication. Results will also be reported on ClinicalTrials.gov in accordance with regulatory requirements. No publication restrictions apply.

## Discussion

This study aims to provide a novel approach to preventing RGC dysfunction, a defining feature of glaucoma. To our knowledge, the IVMGS trial represents the first investigation of the effects of specific 1CM-based supplements (B_6_, B_9_, B_12_, and choline) on retinal function in glaucoma patients. The use of ffERG as the primary endpoint provides an objective, global measure of RGC function, thereby offering valuable data for evaluating the neuroprotective potential of 1CM supplemention. Given glaucoma’s characteristically slow progression, ffERG’s potential capability in detecting changes in the inner retina makes it suitable in a research setting for evaluating treatment efficacy during a 1-year period.

However, translating results from preclinical rodent models to human glaucoma presents challenges. This study builds on prior research in rodent models of glaucoma, which differ from the pathophysiology of glaucoma in humans. In rodent models, experimentally induced rapid IOP increases lead to RGC loss, whereas human glaucoma progresses more slowly and is influenced by factors like aging, genetics, and environmental factors that are not fully replicated in the preclinical animal models used. Additionally, the trial’s generalizability is limited by its exclusion criteria. Only patients with mild to moderate glaucoma and normal IOP are included, excluding patients with advanced disease or prior IOP-lowering surgery. These patients are excluded because neuroprotective therapies aim to preserve remaining retinal ganglion cells, and significant damage makes it harder to detect treatment effects. Moreover, the 12-month window may not fully capture the long-term, supposed neuroprotective effects of 1CM-based supplementation on glaucoma progression, since the disease normally progresses slowly.

In conclusion, the IVMGS trial addresses a critical gap in glaucoma treatment by exploring the neuroprotective potential of a 1CM-based supplementation. If proven effective, this supplementation could offer a low-cost, accessible neuroprotective strategy to complement existing glaucoma therapy.

## Trial status

Recruitment started in March 31, 2025, and is expected to be completed by March 31, 2026. The estimated study completion date is March 31, 2027.

## Supplementary Information

Supplementary Material 1.

Supplementary Material 2.

## Data Availability

Access to the final dataset will be restricted to the PI (RB) and the Research Data Office (RDO) at Karolinska Institute. Inquiries and requests for access to the dataset will be handled by the RDO at Karolinska Institute.

## Abbreviations

RGCs: Retinal ganglion cells
IOP: Intraocular pressure
NAD: Nicotinamide adenine dinucleotide
IVMGS: Interventional Vitamin Mix Glaucoma Study
1CM: One-carbon metabolism
POAG: Primary open-angle glaucoma
PEX: Pseudoexfoliation glaucoma
ERG: Electroretinography
PhNR: Photopic negative response
RNFL: Retinal nerve fiber layer
OCT: Optical coherence tomography
VA: Visual acuity
GCL: Ganglion cell layer
IPL: Inner plexiform layer
Vmax: Maximum saturating amplitude
MD: Mean deviation
PSD: Pattern standard deviation
VFI: Visual Field Index
OHT: Ocular hypertension
GPA: Guided Progression Analysis
BP: Blood pressure
HR: Heart rate
REDCap: Research Electronic Data Capture
GAT: Goldmann Applanation Tonometry
ETDRS: Early Treatment Diabetic Retinopathy Study
SD-OCT: Spectral-Domain Optical Coherence Tomography
PBMCs: Peripheral blood mononuclear cells
5-mC: 5-Methylcytosine
5-hmC: 5-Hydroxymethylcytosine
PCA: Principal component analysis
PLS-DA: Partial Least Squares Discriminant Analysis
NTG: Normal tension glaucoma
PXFG: Pseudoexfoliation glaucoma
LINE-1: Long Interspersed Nucleotide Element 1
