# Supplementary material for: Interventional Vitamin Mix Glaucoma Study (IVMGS): study protocol for a prospective, randomized, two-arm, single-center trial in existing glaucoma patients
Source: Trials. 2025 Oct 13;26:403. doi: 10.1186/s13063-025-09168-z (PMC12516856; doi:10.1186/s13063-025-09168-z)
Supplement: Supplementary file 1 — Supplementary Material 1. [file 13063_2025_9168_MOESM1_ESM.docx]

**Forskningspersonsinformation**

Vi vill fråga dig om du vill delta i forskningsprojektet ”Behandling av glaukom med B-vitamin (B-mix)”? Du tillfrågas eftersom du har ögonsjukdomen glaukom (grön starr). I det här dokumentet får du information om projektet och om vad det innebär att delta.

**Bakgrund och syfte**

Glaukom är en ögonsjukdom som skadar synnerven och ger de upphov till skador i ögats synfält, dvs. skador i det perifera seendet. Det är svårt att själv upptäcka de perifera synfältsskadorna. Glaukom diagnosticeras genom att titta på synnerven genom ögonspegling och numera oftast genom olika datoriserade fotografiska metoder, samt genom att mäta synfältet.

Glaukom ger en mycket varierande sjukdomsbild där många patienter har en långsam och obetydlig försämringstakt medan hos andra är den snabb och betydande. Orsaken till sjukdomen är inte helt känd, men ett förhöjt ögontryck bidrar till utvecklingen av skadorna. Att sänka ögontrycket är hittills den enda behandling med bevisad effekt på sjukdomsförloppet. Behandling är inte botande, men minskar risken för ytterligare skador. Många patienter fortsätter dock försämras trots ögontrycksänkning och en svensk studie har visat att drygt 40% av glaukompatienter blir blinda på ena ögat under sin livstid trots ögontryckssänkande behandling. Därför söker man efter andra möjligheter att bromsa skadeutvecklingen.

Nya rön har påvisat att personer med glaukom har sänkt koncentration av b-vitamin i blod jämfört med friska individer. Vidare har djurstudier visat att metabola förändringar i näthinnans nervceller kan utgöra en del av orsaken till glaukomsjukdomen. Vår forskargrupp har i djurmodell nyligen funnit att kosttillskott med B-mix (en kombination av olika b-vitaminer) bromsar dessa nedbrytande metabola processer i nervcellerna och därigenom skyddar synnerven. Detta utgör en ny angreppspunkt för att behandla sjukdomen. B-vitaminer har i en pilotstudie på glaukompatienter visat att ha en positiv effekt på synfunktionen. B-mix har dokumenterat få biverkningar efter decennier av kliniskt användande vid t.ex. typ I diabetes. B-mix borde därmed vara en lämplig kandidat för att behandla glaukomsjukdomen.

Det huvudsakliga syftet med vår forskningsstudie är att undersöka om behandling med B-mix är effektiv vid glaukom. Vi vill alltså undersöka om försämringstakten skiljer sig hos glaukompatienter som får B-mix-behandling med dem som inte behandlingen.

**Hur går studien till?**

Detta är en 12 månaders studie. Du erhåller samma behandling under hela perioden. Du som tillfrågas om deltagande i studien behandlas redan trycksänkande för din öppenvinkelglaukom eller pseudoexfoliationsglaukom. Om du accepterar att delta kommer vi att kalla dig till ett besök där vi utför vanliga ögonundersökningar såsom synskärpa, synfält och ögontrycksmätning. Vi kommer även att mäta synnervens funktion med ERG (elektroretinogram) genom att mäta ström som bildas när ögat utsätts för ljusblixt. En tunn elektrod placeras under nedre ögonlock och avläser svaret. Undersökningen är inte smärtsam. Dessutom kommer vi mäta puls och blodtryck och fråga om aktuella mediciner och fråga om vissa riskfaktorer, såsom ärftlighet för glaukom, samt ta blodprov. Därefter kommer du att lottas till en av två behandlingsgrupper;

1. Behandling med B-mix (totalt 5 tabletter: 1 tabletter med 25 mg B6, 2 tabletter med 400 µg B9 och 1 mg B12, och 2 tabletter med 500 mg Kolin, per dag på morgon; i 12 månader.

2. Ingen ytterligare behandling än den du redan har idag för din glaukom.

Antalet besök i denna studie är fler än vid normal uppföljning av glaukom för att snabbt kunna upptäcka en oacceptabel försämringstakt. Det kan bli fler besök om behandlande läkare anser att det skulle behövas. Besöksplanen ser ut på följande sätt:

Besök 0 – studien och eventuell behandling inleds

Besök 1 – 3 månader efter att du inkluderades i studien

Besök 2 – 6 månader efter att du inkluderades i studien

Besök 3 – 9 månader efter att du inkluderades i studien

Besök 4 – 12 månader efter att du inkluderades i studien (studieavslut)

**Möjliga risker med att delta i projektet?**

B-mix har dokumenterat få biverkningar och har använts i kliniskt bruk under lång tid så vi förväntar oss inte att allvarliga biverkningar tillstöter. Det går dock inte att utesluta att B-mix ger biverkningar och därför frågas alla deltagare om potentiella biverkningar vid varje besök. Undersökningar som tillämpas i detta projekt är rutinundersökningar inom glaukomvård förutom ERG samt blodprov. ERG undersökningen medför ljusblixtar en kort stund men innebär ingen risk för ögat. Blodprovstagning är en rutinåtgärd inom sjukvården och kan av vissa upplevas som något obehaglig men är ofarligt.

**Finns det några fördelar?**

Om B-mix visar sig vara effektivt vid glaukombehandling är det fördelaktigt för de patienter som får behandlingen. I övrigt inga speciella fördelar, mer än en noggrann kontroll av utvecklingen av glaukomutvecklingen samt att tidsplan för besöken kommer att följas striktare än vad som idag ofta inte är möjligt på grund av vårdköer.

**Vad händer med mina uppgifter?**

Alla uppgifter kommer att databehandlas, analyseras och redovisas på gruppnivå, men kommer inte kunna spåras till dig som person. Du har rätt till radering av dina uppgifter enligt EU:s dataskyddsförordning. Dock finns ett undantag för forskning som kan begränsa den rättigheten och detta prövas vid varje enskild begäran. Du har även rätt till att klaga till Datainspektionen vid felaktig hantering av personuppgifter enligt GDPR. Forskningshuvudmannen, Karolinska Institutet, ansvarar för behandlingen av personuppgifter (kontakt: [dataskyddsombud@ki.se](mailto:dataskyddsombud@ki.se)).

Dina uppgifter kommer att behandlas på ett sådant sätt att ingen obehörig person får tillgång till dem. Du kan även begära att felaktiga uppgifter om dig rättas. Projektet är granskat och godkänt av Etikprövningsmyndigheten.

**Hur får jag information om studiens resultat?**

Du får fortlöpande under studiens gång information om dina egna undersökningsresultat på samma sätt som om du varit en ”vanlig” patient. Resultat av hela studien kommer att presenteras i internationell vetenskaplig tidskrift. Patientföreningen ”Svenska Glaukomförbundet” har visat intresse för studien och sannolikt kommer resultaten presenteras vid något av deras möten samt i deras medlemsblad ”Ögontrycket”

**Biobanksprover**

Dina prov kommer att hanteras i enlighet med Biobankslagen (2023:38). Vid tre olika tillfällen (vid inklusion, samt efter 3 och 9 månader) tas blodprov. Vid varje tillfälle tas två rör om 10 ml blod vardera, vilket innebär totalt 20 ml blod.

Provet märks med en kod som ger information om ålder, kön, typ av prov och sjukdomsgrupp (primärt öppenvinkelglaukom eller pseudoexfoliationsglaukom). Kodnyckeln, som kan koppla ihop dina personuppgifter med provet, förvaras på den provtagande kliniken i ett säkert skåp som endast behörig personal har tillgång till. Kodnyckeln hanteras så att ingen obehörig kan få del av den.

Blodproverna kommer att inrättas och förvaras i Stockholms Medicinska Biobank (registreringsnummer 914). Efter att analyserna är genomförda kommer proverna antingen att förstöras eller fortsätta att förvaras i biobanken i högst ett år efter studiens avslut, beroende på det samtycke du har lämnat. Om samtycke har dragits tillbaka kan redan insamlade data (t.ex. analyssvar) som erhållits före tillbakadragandet av samtycket fortsätta att användas.

**Försäkring, ersättning**

Som vid all sjukvård så omfattas du av patientskadeförsäkringen och det utgår inte någon ekonomisk ersättning för deltagande. Besöken kommer dock att vara avgiftsfria.

**Deltagande**

Ditt deltagande är helt frivilligt. Du kan avstå från att vara med i studien utan att det påverkar din framtida behandling. Du kan när som helst avbryta ditt deltagande utan att behöva förklara varför. Ingen ytterligare information kommer då att inhämtas.

För ytterligare information eller frågor, är du välkommen att kontakta undertecknande.

Rune Brautaset

Professor

Karolinska Institutet

Tel: xxxx xxx xxx

**Skriftligt samtycke för deltagande i ”Behandling av glaukom med B-vitamim (B-mix)”**

• Jag har tagit del av både muntlig och skriftlig information om ”Behandling av glaukom med B-vitamin (B-mix)”, fått tillfälle att ställa frågor och fått dem besvarade.

• Jag samtycker att deltaga i forskningsprojektet ” Behandling av glaukom med B-vitamin (B-mix)”.

• Jag samtycker att uppgifter om mig behandlas på det sätt som det beskrivs i informationen om ”Behandling av glaukom med B-vitamin (B-mix)”.

• Jag samtycker till att mitt biologiska prov bevaras i högst ett år efter studiens slut.

Jag samtycker:

För- och efternamn …………………………………………………………………………………………

Datum ……………………………………..

Namnteckning ………………………………………………………………………………………..

**Klinikens underskrift**

Jag bekräftar att patienten mottagit muntlig och skriftlig information om ”Behandling av glaukom med B-vitamin (B-mix)” projektet:

För- och efternamn …………………………………………………………………………………………

Datum ……………………………………..

Namnteckning ………………………………………………………………………………………..
